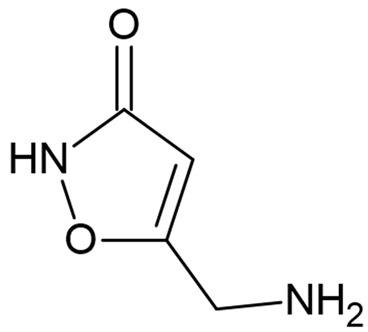# Therapeutic Potential of Complementary and Alternative Medicines in Peripheral Nerve Regeneration: A Systematic Review

**DOI:** 10.3390/cells10092194

**Published:** 2021-08-25

**Authors:** Yoon-Yen Yow, Tiong-Keat Goh, Ke-Ying Nyiew, Lee-Wei Lim, Siew-Moi Phang, Siew-Huah Lim, Shyamala Ratnayeke, Kah-Hui Wong

**Affiliations:** 1 Department of Biological Sciences, School of Medicine and Life Sciences, Sunway University, Petaling Jaya 47500, Malaysia; tiongkeatgoh@gmail.com (T.-K.G.); kynyiew@gmail.com (K.-Y.N.); shyamalar@sunway.edu.my (S.R.); 2 Neuromodulation Laboratory, School of Biomedical Sciences, Li Ka Shing Faculty of Medicine, The University of Hong Kong, 21 Sassoon Road, L4 Laboratory Block, Hong Kong; 3 Institute of Ocean and Earth Sciences, Universiti Malaya, Kuala Lumpur 50603, Malaysia; phang@um.edu.my; 4 Faculty of Applied Sciences, UCSI University, Cheras, Kuala Lumpur 56000, Malaysia; 5 Department of Chemistry, Faculty of Science, Universiti Malaya, Kuala Lumpur 50603, Malaysia; shlim80@um.edu.my; 6 Department of Anatomy, Faculty of Medicine, Universiti Malaya, Kuala Lumpur 50603, Malaysia

**Keywords:** complementary and alternative medicines, natural products, peripheral nerve injury, nerve repair, nerve regeneration, functional recovery

## Abstract

Despite the progressive advances, current standards of treatments for peripheral nerve injury do not guarantee complete recovery. Thus, alternative therapeutic interventions should be considered. Complementary and alternative medicines (CAMs) are widely explored for their therapeutic value, but their potential use in peripheral nerve regeneration is underappreciated. The present systematic review, designed according to guidelines of Preferred Reporting Items for Systematic Review and Meta-Analysis Protocols, aims to present and discuss the current literature on the neuroregenerative potential of CAMs, focusing on plants or herbs, mushrooms, decoctions, and their respective natural products. The available literature on CAMs associated with peripheral nerve regeneration published up to 2020 were retrieved from PubMed, Scopus, and Web of Science. According to current literature, the neuroregenerative potential of *Achyranthes* *bidentata*, *Astragalus* *membranaceus*, *Curcuma* *longa*, *Panax* *ginseng*, and *Hericium* *erinaceus* are the most widely studied. Various CAMs enhanced proliferation and migration of Schwann cells *in vitro*, primarily through activation of MAPK pathway and FGF-2 signaling, respectively. Animal studies demonstrated the ability of CAMs to promote peripheral nerve regeneration and functional recovery, which are partially associated with modulations of neurotrophic factors, pro-inflammatory cytokines, and anti-apoptotic signaling. This systematic review provides evidence for the potential use of CAMs in the management of peripheral nerve injury.

## 1. Introduction

Peripheral nerve injury (PNI) can result in partial or total loss of motor, sensory and autonomic functions at denervated regions, leading to temporary or life-long disability [1]. In addition to reduced quality of life, functional deficits from PNI have a substantial economic impact on the affected individuals [2]. A recent study found that, over nine years (from 2009 to 2018), more than 550,000 individuals were afflicted by PNI in the United States. Moreover, the incidence rate has more than doubled throughout that period of time [3]. Such injuries are primarily due to vehicular and traumatic accidents, lacerations, and iatrogenic causes [4,5,6].

Despite progressive advances in our understanding of the processes and mechanisms of nerve injury, effective nerve repair and regeneration approaches that ensure complete functional recovery remain scarce [7]. Nerve autograft is considered the gold standard for repairing peripheral nerve defects [8]. However, this method is restricted by limited donor nerves and donor site morbidity, while successful recovery rates remain unsatisfactory [9]. Consequently, alternative strategies for enhancing nerve repairs have been proposed, including the application of nerve conduits and the addition of growth factors [10,11]. Likewise, the exploration of novel therapeutics, even combinatorial therapies, capable of enhancing axonal regeneration and promoting functional recovery, are of great interest.

PNI often results in neuropathic pain, and when conventional treatments are inadequate in providing relief, patients may turn to complementary and alternative medicines (CAMs), such as herbal medicines and nutritional supplements [12]. Indeed, medicinal plants, including the *Acorus calamus* [13], *Curcuma longa* [14], and *Ginkgo biloba* [15], have displayed ameliorating effects in animal models of neuropathic pain. Research on the potential of medicinal plants in the treatment of PNI is prompted by the notion that plants are great sources of natural products (NPs), which are small molecules produced by living organisms. Many NPs are the focus of drug development, as it is generally believed that they are largely devoid of adverse effects compared to synthetic drugs [16,17]. NPs also have the advantage of being evolutionary-driven, thus they are more likely to possess tremendous chemical and structural diversity that facilitates efficient engagement with biologically relevant targets and receptors, making them more biologically active [18]. In fact, many small-molecule drugs that have been approved by regulatory agencies were derived from natural sources [19], including Taxol from *Taxus brevifolia* [20] and Vinblastine from *Catharanthus roseus* [21].

However, compared to the extensive research on naturally derived products for other non-communicable and infectious diseases, NPs remain largely unexplored in the field of nerve repair and regeneration. A review published nearly half a decade ago has shed light on the neuroprotective effects of NPs in PNI models [22]. This review presents current research findings and evaluates the role of CAMs, focusing on plants or herbs, mushrooms, and decoctions, as well as their NPs, in peripheral nerve regeneration, to highlight their therapeutic potential for the management of PNI.

## 2. Materials and Methods

This systematic review was designed according to guidelines of Preferred Reporting Items for Systematic Review and Meta-Analysis Protocols (PRISMA-P) [23].

### 2.1. Search Strategy and Data Extraction

A literature search was performed to find all relevant publications up to 25 October 2020 across three electronic databases, PubMed, Scopus, and Web of Science. The following keywords were used to search each respective database: ((“peripheral* nerve* regenera*” OR “peripheral* nerve* repair*” OR “neuroregenera*”) AND (“alga*” OR “seaweed*” OR “plant” OR “natural product*” OR “mushroom” OR “Basidiomycete*” OR “herb*” OR “Traditional Chinese Medicine*” OR “alternative medicine” OR “complementary medicine*”)).

### 2.2. Eligibility Criteria

Research articles describing the use of plants or herbs, mushrooms, algae, decoction, and their natural products in peripheral nerve repair and regeneration, written in English, and having full-text availability were considered. Articles not representing original research studies and NPs derived from sources other than plants, herbs, algae, and mushrooms were excluded (e.g., *Lumbricus rubellus*—earthworm). Retrieved articles were screened based on their title, abstract, and full-text to determine their eligibility for inclusion in this review.

## 3. Results

**Figure 1:**
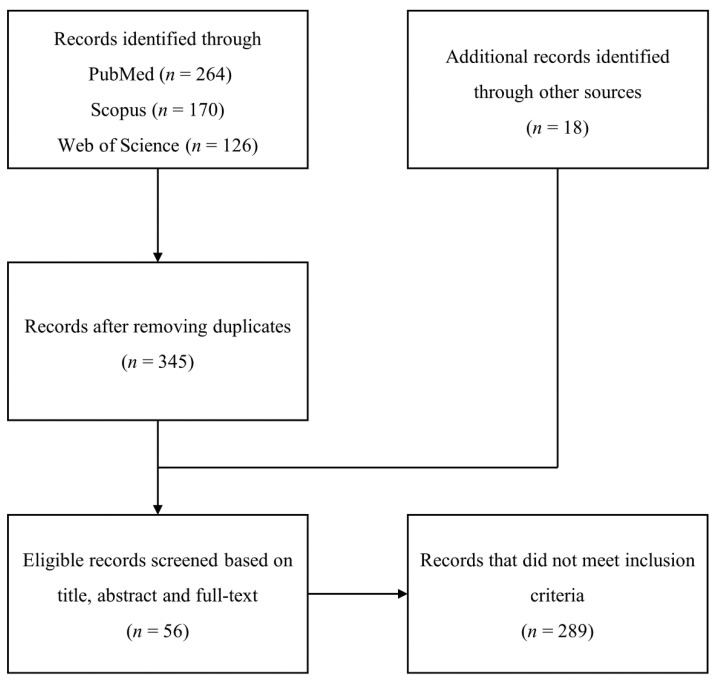
Flow diagram of the literature search procedure for the selection of studies up to 25 October 2020 on the use of plants, mushrooms, algae, decoctions, and their natural products (NPs) in peripheral nerve repair and regeneration. Only articles written in English, and having full-text availability were included. Articles not representing original research studies and NPs derived from sources other than plants, herbs, algae, and mushrooms were excluded.

A preliminary search across the three databases yielded 560 records, of which 215 were duplicates (Figure 1). Together with 18 other records identified by other means, the remaining articles were screened based on the eligibility criteria, resulting in 289 additional records being excluded, leaving 56 records remaining and their findings being included in the qualitative synthesis (Figure 1). The studies investigated the neuroregenerative potential of 25 species of plants, three different mushrooms, and four traditional Chinese medicine decoctions, of which 18 known NPs were characterized. None of the studies investigated the potential of algae in peripheral nerve regeneration.

Among the 58 records, the majority of the reported findings were from *in vivo* studies (38 records) that used mainly histological and electrophysiological evaluation to examine peripheral nerve regeneration in rat models of sciatic nerve injury (SNI). In contrast, 11 records were *in vitro* studies, which included reports of the promoting effects of plants, mushrooms, decoctions, and their natural products on the proliferation and migration of Schwann cells (SCs), and on neurite outgrowth in dorsal root ganglion (DRG) explants and neurons. Additionally, nine records included both *in vitro* and *in vivo* studies. In terms of the mechanisms of the biological effects, regulation of the mitogen-activated protein kinase (MAPK) pathway was reported to be highly involved across these studies.

## 4. Discussion

### 4.1. Current Therapeutic Approaches against Peripheral Nerve Injuries

Peripheral nerves are prone to injury because of their delicate structures and superficial location throughout the human body. The prevalence of PNI together with its societal impact poses a health concern that needs to be addressed properly. Current treatment strategies for PNI are divided into surgical and non-surgical approaches that can be effective when applied appropriately [24]. Surgical techniques, including suturing of severed nerves and nerve grafting, do yield successful outcomes but are sometimes not feasible due to limitations such as the timing of surgery, size of nerve gaps, and donor site morbidity [25,26]. Consequently, other promising alternatives have emerged in recent years and have been receiving increasing attention, such as the utilization of different nerve conduits capable of housing and delivering biological cues whilst enhancing and guiding nerve regeneration 11, growth factor treatments [27], and cell-based therapies [28]. In contrast, non-surgical options for the management of PNI are far more limited, including approved medications on the market, electrical nerve stimulation [29], and the application of phytochemicals and secondary metabolites. The latter is widespread in other areas of research including cancer [30] and neurological disorders [31], but are far less prevalent in the field of peripheral nerve regeneration.

### 4.2. Mechanisms of Peripheral Nerve Injury and Regeneration

**Figure 2:**
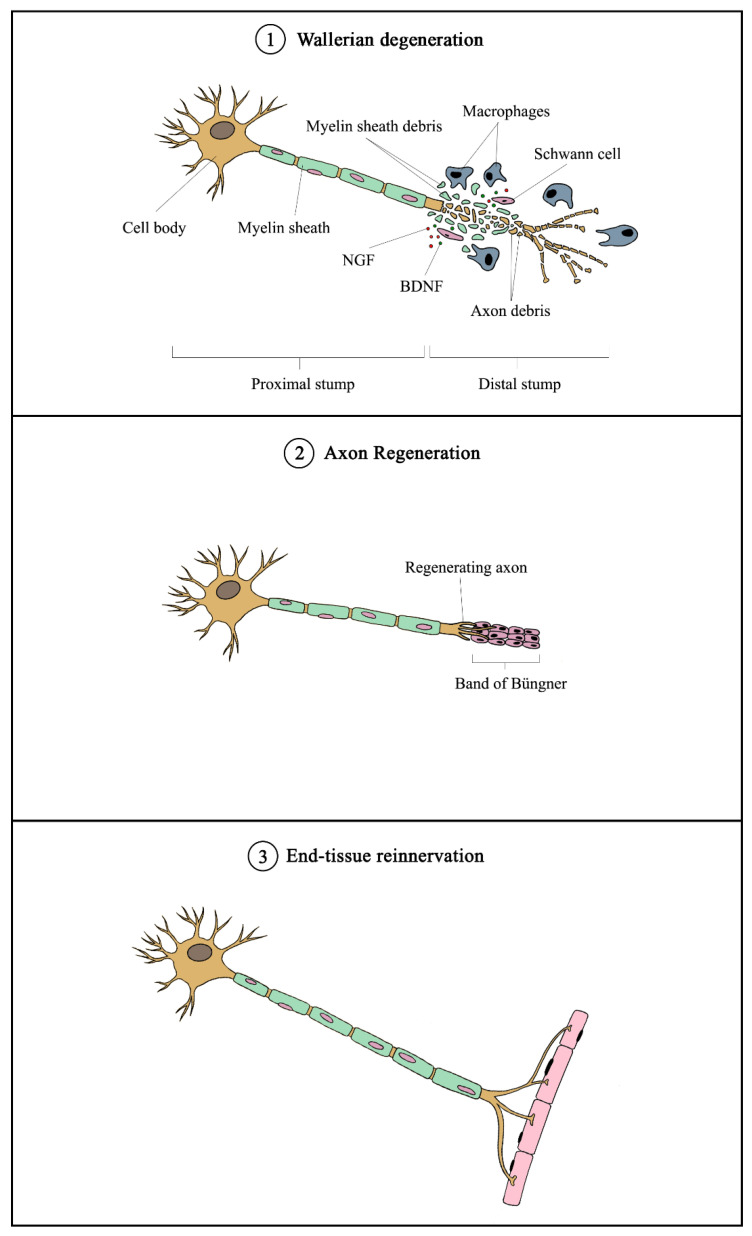
Overview of mechanism of peripheral nerve injury and regeneration. Following nerve injury, Wallerian degeneration occurs, in which axons begin to disintegrate at the distal end, and growth factors (such as NGF and BDNF) are released by Schwann cells. Galectin-3 macrophages are recruited to remove axonal debris and degrade myelin sheaths. Subsequently, SCs align to form the Band of Büngner, which guides the regenerating axons from the proximal to distal sites. Eventually, the regenerated axons innervate the end tissue to complete the recovery process. NGF—nerve growth factor; BDNF—brain-derived neurotrophic factor.

Nerve bundles are primarily composed of axons covered with myelin sheaths produced by Schwann cells with fibroblasts scattered in between the nerve fibers. During peripheral nerve injury, instantaneous tissue damage occurs at the site of the lesion together with the accumulation of galectin-3 macrophages, whereas nerve stumps that are distally located undergo cellular variation despite not being directly affected [32]. After an axonal injury, Wallerian degeneration occurs, followed by axonal regeneration, and eventually end-organ reinnervation (see Figure 2) [33]. Wallerian degeneration takes place 24 to 48 h following nerve injury. Axons begin to disintegrate and growth factors such as nerve growth factor (NGF) and brain-derived neurotrophic factor (BDNF) are released by SCs in the segment distal to the injured site. Galectin-3 macrophages are then recruited to the distal end, which contributes to myelin degradation and removal of remaining debris [34]. Growth factors are also retrogradely transported proximally toward the cell body. Subsequent removal of deteriorated myelin and axonal matter leads to the proliferation and alignment of SCs, forming the bands of Büngner that further guide the regenerating axons from the proximal to the distal site [35]. Axonal regeneration in humans is known to occur at a rate of approximately 1 mm per day [36], which would require months or even years for severe nerve injuries to fully recover. Moreover, poor functional recovery can occur due to a number of reasons, including progressive failure of axonal regeneration, disruption of SC function in providing a growth-supportive environment, and misdirection of regenerating axons [36].

### 4.3. Role of Schwann Cells in Nerve Regeneration

**Figure 3:**
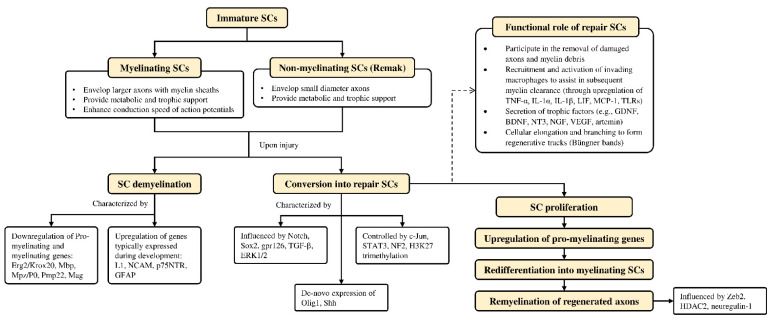
Overview of Schwann cell plasticity and their roles following peripheral nerve injury. Immature SCs develop into either myelinated or non-myelinated forms depending on the type of axon association. Upon nerve injury, SCs are capable of converting into a repair phenotype alongside the demyelination process that is mediated by different genes and transcriptional mechanisms. These events promote neuronal survival and enhance axonal regeneration following injury. Subsequently, repair SCs can be reprogrammed back to remyelinate regenerated axons. Further details on SC plasticity are presented in the reviews by Jessen & Mirsky [39] and Nocera & Jacob [100]. BDNF—brain-derived neurotrophic factor; Erg2/Krox20—early growth response 2; ERK—extracellular signal-regulated protein kinase; GDNF—glial cell-derived neurotrophic factor; GFAP—glial fibrillary acidic protein; gpr126—adhesion G protein-coupled receptor G6; H3K27—methylation of histone H3 on lysine 27; HDAC2—histone deacetylase 2; IL—interleukin; L1—L1 cell adhesion molecule; LIF—leukemia inhibitory factor; Mag—myelin associated glycoprotein; Mbp—myelin basic protein; MCP-1—monocyte chemotactic protein 1; Mpz/P0—myelin protein zero; NCAM—neural cell adhesion molecule; NF2—neurofibromatosis 2; NGF—nerve growth factor; NT3—neurotrophin-3; Olig1—oligodendrocyte transcription factor 1; p75NTR—p75 neurotrophin receptor; Pmp22—peripheral myelin protein 22; SCs—Schwann cells; Shh—Sonic Hedgehog; Sox2—(sex determining region Y)-box 2; STAT3—signal transducer and activator of transcription 3; TGF-β—transforming growth factor-β; TLRs—Toll-like receptors; TNF-α—tumor necrosis factor-α; VEGF—vascular endothelial growth factor; Zeb2—zinc finger E-box-binding homeobox 2.

Schwann cells are supportive glial cells that are known to play a pivotal role in the proper functioning and maintenance of peripheral nerves. They are responsible for producing the basal lamina that determines the polarity of SCs and myelinating axons [37]. The myelin sheaths on axons allow the conduction of action potentials at high velocity via the formation of specialized nodes of Ranvier [38]. The high plasticity of SCs allows them to further develop into repair phenotypes in response to nerve injury (Figure 3). Following nerve injury, SCs can re-differentiate into repair SCs that align themselves to form bands of Büngner. This in turn allows axons to emerge from growth cones proximal to the injured site, which then elongate along the bands until the target organ is reinnervated. The repair SCs also participate in the removal of axon and myelin debris, and they can recruit macrophages to assist in the process [39]. In addition, repair SCs can also secrete neurotrophic factors that help promote cellular survival, proliferation, and differentiation, which are all essential for peripheral nerve repair [40]. Due to the importance of SCs in promoting peripheral nerve regeneration, it is expected that any disruption in SC proliferation, such as that caused by impairment in cyclin D1, will affect nerve regeneration following injury [41]. However, findings from past studies suggest that axonal regeneration is independent of SC proliferation [42,43]. Nevertheless, considering the association of SCs with axonal elongation and myelination, it is reasonable to hypothesize that enhanced SC proliferation may lead to greater regenerative potential. Hence, numerous studies have attempted to investigate the effects of NPs in promoting the proliferation and migration ability of SCs (Table 1).

**Table 1:** Summary of plants, mushrooms, and decoctions their natural products relating to peripheral nerve regeneration.

Source	Molecule(s)/Ingredients	Experimental Model	Effective Concentration	Application Method	Biological Effect	Mechanism	Reference
PLANT							
*Achyranthes bidentata*	Polypeptides	*In vitro* (SCs isolated from the sciatic nerves of 1-day old SD rats)	0.1 µg/mL	Incubation	Promoted migration of SCs	Upregulation of NOX4/DUOX2-derived ROS production	[44]
	*Polypeptides*	*In vitro* *(DRG explants harvested from spinal and peripheral roots of postnatal day 1 SD rats)*	0.01, 0.1, 1 µg/mL (dose-dependent manner)	Incubation	Promoted neurite outgrowth from cultured DRG explants/neurons	Activation of ERK1/2	[45]
		*In vivo* (Adult New Zealand rabbits)	6.0 mg/kg	Intravenous injection	Enhanced nerve regeneration and functional restoration after crush injury to rabbit common peroneal nerve (increased CMAP, density, diameter and thickness of myelinated fibers, and number of motor neurons in anterior horn)	N/A	
	Polypeptides (Fraction K)	*In vitro* (DRG explants harvested from spinal and peripheral roots of postnatal day 1 SD rats)	50, 250 ng/mL (dose-dependent manner)	Incubation	Promoted neurite outgrowth in DRG explant and neurons	Activation of ERK1/2	[46]
		*In vivo* (ICR mice)	10 mg/kg	Intravenous injection	Promoted peripheral nerve regeneration in mice after SNI (increased diameter and thickness of myelinated fibers, CSA of gastrocnemius muscle fibers, SFI, and CMAP)	N/A	
	Polypeptides	*In vivo* (SD rats)	2 mg in 0.2 mL saline	Intraperitoneal injection	Promoted functional and histological recovery after rat sciatic nerve crush (increased SFI, CMAP, MNCV, myelin thickness, lamellae number, CSA of gastrocnemius muscle fibers)	Modulation of mRNA expression of GAP-43, neurotrophic factors (NGF, BDNF, CNTF), and neurotrophic factor receptors (TrkA, TrkB)	[47]
	Polypeptides	*In vivo* (ICR mice)	1, 4, 16 mg/kg (dose-independent manner)	Tail vein injection	Promoted functional and histological recovery after rat sciatic nerve crush (increased SFI, CMAP, MNCV, number, and diameter of myelinated fibers, axon diameter, myelin thickness, lamellae number, CSA of gastrocnemius muscle fibers)	N/A	[48]
	Aqueous extract	*In vivo* (Adult New Zealand rabbits)	10, 20 mg/kg (dose-dependent manner)	Intravenous injection	Promoted peripheral nerve regeneration in the crushed common peroneal nerve in rabbits (increased CMAP, CSA of tibialis posterior muscle, number of regenerated myelinated nerve fibers, and motoneurons in anterior horn of the spinal cord)	N/A	[49]
Alpinate Oxyphyllae Fructus (*Alpinia oxyphylla* Miq)	Protocatechuic acid	*In vitro* (RSC96 SCs)	1 mM	Incubation	Promoted proliferation and survival of RSC96 SCs	Upregulation of IGF-1 and activation of PI3K/Akt signaling	[50]
	Aqueous extract	*In vitro* (RSC96 SCs)	Proliferation: 20, 60, 200 µg/mL (dose-independent manner Migration: 20–200 µg/mL (dose-dependent manner	Incubation	Promoted proliferation and migration of RSC96 SCs	Upregulation of PAs (uPA, tPA) and MMP2/9 mediated through the activation of MAPK pathway (ERK1/2, JNK, p38)	[51]
		*In vivo* (SD rats)	30, 60, 100, 150, 200 µg/mL (dose-independent manner)	Injection into a silicone rubber tube bridging a 15mm sciatic nerve defect	Promoted peripheral nerve regeneration in rats with SNI		
*Astragalus membranaceus*	Astragaloside IV	*In vivo* (BALB/c mice)	2.5, 5, 10 mg/kg (dose-dependent manner)	Intraperitoneal injection	Promoted sciatic nerve regeneration and functional recovery in mice (increased number and diameter of myelinated nerve fibers, MNCV, CMAP)	Upregulation of GAP-43 expression	[52]
	Astragaloside IV	*In vivo* (SD rats)	50 µM	Injection into a silicone rubber tube bridging a 15mm sciatic nerve defect	Promoted peripheral nerve regeneration in rats with SNI (increased number of myelinated axons and CMAP)	N/A	[53]
	Extract	*In vivo* (SD rats)	3 g/kg in 0.01 M of PBS	Intragastric gavage	Promoted peripheral nerve regeneration in rats with SNI (increased MNCV and latency, fluorogold labeling in the DRG, mean axonal density, percentage of CGRP area ratio, and macrophage density)	Modulation of local growth factors (FGF, NGF, PDGF, TGF-β) and immunoregulatory factors (IL-1, IFN-γ)	[54]
	Aqueous extract	*In vitro* (RSC96 SCs)	Proliferation: 12.5, 125, 250, 500 µg/mL (optimal at 12.5 µg/mL) Migration: 1.25, 12.5, 125, 250, 500 µg/mL (optimal at 1.25 µg/mL)	Incubation	Promoted proliferation and migration of RSC96 SCs	Proliferation: Increased cyclin protein A, D1, and E via ERK and p38 signaling pathways Migration: Activation of FGF-2 signaling, leading to upregulation of uPA and downregulation of PAI-1	[55]
*Centella asiatica*	Hydro-ethanolic extract	*In vivo* (SD rats)	400 µg/mL	Nerve conduit developed using decellularized artery seeded with *C. asiatca*-neurodifferentiated mesenchymal stem cells bridging a 15mm sciatic nerve defect	Promoted nerve regeneration and functional restoration in rats with SNI (increased CMAP, latency, MNCV, confirmation of angiogenesis, increased MBP expression, and number of myelinated axons)	N/A	[56]
*Citrus medica* var. *sarcodactylis*	Aqueous extract	*In vitro* (RSC96 SCs)	0.85, 1.7, 2.55, 3.4, 4.25 µg/mL (dose-dependent manner)	Incubation	Promoted proliferation and migration of RSC96 SCs	Proliferation: Upregulation of cyclin A and B1 Migration: Activation of FGF-2 signaling, leading to the upregulation of uPA and MMP-9	[57]
*Codonopsis pilosula*	Aqueous extract	*In vitro* (RSC96 SCs)	20, 40, 60, 80, 100 µg/mL (dose-independent manner)	Incubation	Promoted proliferation and migration of RSC96 SCs	Proliferation: Enhanced IGF-I signaling pathway, cell cycle controlling protein expressions (cyclin A, D1, E) and MAPK pathway (ERK, p38) Migration: Stimulated FGF-2-uPA-MMP9 migration pathway	[58]
*Crocus sativus*	Crocin	*In vivo* (Wistar rats)	20, 80 mg/kg	Intraperitoneal injection	Promoted functional recovery in rats with SNI (Increased SFI, reduced plasma MDA levels, alleviated histological changes due to a crushing injury)	N/A	[59]
*Curcuma longa*	Alcoholic extract	*In vivo* (Wistar rats)	100 mg/kg (3, 6, or 9 times across 28 days)	Intraperitoneal injection	Protected against peripheral nerve degeneration in rats with SNI (Increased number of intact neurons in the right ventral horn of spinal cord region)	N/A	[60]
	Curcumin	*In vivo* (SD rats)	100 mg/kg (dissolved in olive oil)	Oral gavage	Promoted peripheral nerve regeneration in rats with SNI (increased mean cell volume, total volume and surface of DRG cells, total number, diameter, and area of myelinated nerve fibers)	N/A	[61]
	Curcumin	*In vivo* (SD rats)	100 mg/kg (dissolved in olive oil)	Oral gavage	Promoted functional recovery (improved SFI) and protective effect on DRG (increased volume and number of A- and B- cells, number of satellite cells) in rats with SNI	N/A	[62]
	Curcumin	*In vivo* (SD rats)	50, 100, 300 mg/kg	Intraperitoneal injection	Promoted peripheral nerve regeneration in rats with SNI (increased number of motoneurons, number and diameter of myelinated axons, SFI, MNCV, amplitude of CMAP, muscle fiber area and reduced latency of CMAP, mechanical withdrawal threshold, thermal withdrawal latency)	N/A	[63]
	Curcumin	*In vitro* (SCs isolated from S100β-DsRed transgenic mice)	0.04-1 µM (0.1 µM having the highest proliferative effect)	Incubation	Promoted proliferation and migration of SCs	Proliferation: Modulated by ERK and p38 kinase pathways	[64]
*Curcuma longa* (curcumin); from honeybees (propolis)	Curcumin, propolis	*In vivo* (Wistar rats)	Curcumin (100 mg/kg) Propolis (200 mg/kg)	Administration through a nasogastric tube	Promoted functional recovery in rats with SNI (Increased SFI and amplitude of CMAP, reduced latency time)	N/A	[65]
*Dioscoreae rhizoma*	Aqueous extract	*In vivo* (SD rats)	10 mg/mL	Applied directly into the crush site	Promoted peripheral nerve regeneration in rats with SNI (increased number of DRG sensory neurons and motor neurons in the spinal cord)	Increasing protein levels of GAP-43 and Cdc2	[66]
*Epimedium*	Icariin	*In vivo* (SD rats)	20 mg in 5 mL	Injection into a poly(lactic-co-glycolic acid) biological conduit sleeve bridging a 5mm sciatic nerve defect	Promoted peripheral nerve regeneration in rats with SNI (increased sciatic nerve conduction velocity and number of myelinated fibers)	N/A	[67]
	Epimedium extract, icariin	*In vivo* (SD rats)	4.873 mg/mL	Intragastric administration	Promoted peripheral nerve regeneration in rats with SNI (Increased SFI, nerve regeneration based on nerve pinch test, MNCV, muscle wet weight)	N/A	[68]
*Gardenia jasminoides* Ellis	Genipin	*In vivo* (SD rats)	3% aqueous gelatin solution fixed with 3% genipin	Injection into a silicone rubber tube bridging a 10mm sciatic nerve defect	Promoted peripheral nerve regeneration in rats with SNI	N/A	[69]
*Gastrodia elata* Blume	Gastrodin	*In vitro* (RSC96 SCs)	50, 100, 200 µM (dose-dependent manner)	Incubation	Promoted proliferation of RSC96 SCs in a dose- and time-dependent manner	Inhibition of ERK1/2 phosphorylation and activation of Akt phosphorylation	[70]
*Ginkgo biloba*	*Ginkgo biloba* extract (EGb 761)	*In vivo* (SD rats)	50 mg/kg	Intraperitoneal injection paired with an 18mm acellular nerve allograft bridging a 15mm sciatic nerve defect	Promoted peripheral nerve regeneration in rats with SNI (increased density of regenerated axons, muscle mass, axon number and diameter, expression of CD34 and NF200)	Increasing expression of angiogenesis-related genes (Vegf, Sox18, Prom1, IL-6)	[71]
	*Ginkgo biloba* extract (EGb 761)	*In vitro* (SCs isolated from spinal nerves of 1-day old SD rats)	1, 10, 20, 50, 100 µg/mL (dose-dependent manner)	Incubation	Promoted cell attachment and survival of SCs	N/A	[72]
		*In vivo* (SD rats)	10, 50 µg/mL	Injection into poly(DL-lactic acid-co-glycolic acid) conduit seeded with Schwann cells bridging a 12mm sciatic nerve defect	Promoted histological and functional recovery in rats with SNI (increased number and area of myelinated axons, increased CMAP)		
Ginseng	Ginsenoside Rg1	*In vitro* (RSC96 SCs)	Ginseng: 100, 200, 300, 400, 500 µg/mL Ginsenoside: 5, 10, 15, 20, 25 µg/mL) (Dose-dependent manner for both)	Incubation	Promoted proliferation and migration of RSC96 SCs	Proliferation: Enhancing protein expression of IGF-I pathway regulators (IGF-IR, PI3K, p-Akt, p-Bad, Bcl-2), cell cycle controlling proteins (cyclin D1, E, A), and MAPK signaling pathway (ERK, JNK, p38) Migration: Stimulating the FGF-2-uPA-MMP9 migrating pathway	[73]
	Ginsenoside Rg1	*In vivo*(SD rats)	1.5 mg/kg	Intraperitoneal injection	Promoted peripheral nerve regeneration in rats with SNI (increased number of motoneurons, number, and diameter of myelinated axons, SFI, MNCV, improved CMAP latency and amplitude, the increased average percentage of muscle fiber)	N/A	[74]
	Ginsenoside Re	*In vitro* (SCs isolated from sciatic nerves of 3-day old SD rats)	0.5 mg/mL	Incubation	Promoted proliferation and migration of SCs	Phosphorylation of ERK1/2 and JNK 1/2	[75]
		*In vivo* (SD rats)	2.0 mg/kg	Intraperitoneal injection	Promoted peripheral nerve regeneration in rats with SNI (increased SFI, TSI, PCNA expression level, improved pathological changes due to crushing injury, GAP43, and S-100 expression)		
Green tea	(-)-Epigallocatechin-3-gallate (EGCG)	*In vivo* (Wistar rats)	50 mg/kg	Intraperitoneal injection	Promoted functional recovery (improved outcomes of foot position, toe spreading, extensor postural thrust, hopping reflex, von Frey hair, Randall–Sellito, hotplate, and tail-flick tests), improved morphological recovery in skeletal muscle tissues muscles, and protection towards muscle fibers in rats with SNI	Protection of muscle fibers from cellular death through activation of an anti-apoptotic signaling pathway (modulation of Bax, Bcl-2, and p53 expression)	[76]
	(-)-Epigallocatechin-3-gallate (EGCG)	*In vivo* (Wistar rats)	50 mg/kg	Intraperitoneal injection	Promoted peripheral nerve regeneration in rats with SNI (improved nerve morphology and functional recovery assessed by foot position, extensor postural thrust test, and withdrawal reflex threshold)	Reversal of Bax, Bcl-2, and survivin mRNA expression induced by sciatic nerve injury	[77]
Can be found in a wide variety of plants	Syringic acid	*In vitro* (RSC96 SCs)	600 µM	Incubation	Promoted proliferation and migration of RSC96 SCs	Downregulation of miR-451-5p	[78]
Can be found in a wide variety of plants	Ursolic acid	*In vivo* (BALB/c mice)	2.5, 5, 10 mg/kg (dose-dependent manner)	Intraperitoneal injection	Promoted peripheral nerve regeneration in rats with SNI (increased number and diameter of myelinated nerve fibers and soleus muscle mass)	Increasing S100 protein expression levels	[79]
*Lycium barbarum*	Polysaccharide	*In vitro* (1) PC12 cells (2) Rat SCs (3) DRG neurons isolated from the embryo of 14-day pregnant rat	10, 30, 50 mg/mL (optimal at 30 mg/mL)	Incorporated into core-shell structured nanofibrous scaffolds by coaxial electrospinning	(1) Promoted proliferation and neuronal differentiation of PC12 cells (2) Promoted proliferation and myelination of SCs (3) Promoted neurite outgrowth of DRG neurons	N/A	[80]
Can be found in a wide variety of plants	Quercetin	*In vitro* (RSC96 SCs)	0.1, 1, 10 µg/mL	Incubation	Promoted proliferation of RSC96 SCs	N/A	[81]
		*In vivo* (SD rats)	0.1, 1, 10 µg/mL	Injection into a silicone rubber tube bridging a 15mm sciatic nerve defect	Promoted peripheral nerve regeneration in rats with SNI (increased count and density of myelinated axons, and resulted in larger area and amplitude of CMAP)		
*Morus* sp.	Cortex Mori Radicis (aqueous extract)	*In vivo* (SD rats)	100 mg/kg	Gastrointestinal administration	Reduced blood glucose levels, improved nerve functions (thermal latency and mechanical threshold), reversed the loss of Nissl bodies and induced neurite outgrowth in DRG neurons, and restored the response of growth cones to NGF in diabetic rats	Neurite outgrowth: Increased expression of TRPC1, reduced Ca^2+^ influx, and activation of PI3K/Akt signaling	[82]
*Pueraria lobata*	Puerarin	*In vitro* (RSC96 SCs)	1, 10, 100 µM (dose-independent manner)	Incubation	Promoted growth of SCs	N/A	[83]
		*In vivo* (SD rats)	1, 10, 100 µM (dose-independent manner)	Injection into a silicone rubber tube bridging a 15mm sciatic nerve defect	Promoted peripheral nerve regeneration in rats with SNI (increased density of myelinated axons, CMAP, and MNCV)		
	Serum metabolites (obtained from rats fed with *Pueraria lobata* extract)	*In vitro* (PC12 cells)	0.01, 0.1, 1 unit	Incubation	Enhanced NGF-mediated neurite outgrowth and expression of synapsin I in PC12 cells	N/A	[84]
		*In vivo* (SD rats)	0.01, 0.1, 1 unit	Injection into silicone rubber chamber bridging a 10mm sciatic nerve defect	Promoted peripheral nerve regeneration in rats with SNI (increased mean values of myelinated axon number, endoneurial area, and total nerve area)		
Radix Hedysari	Aqueous extract	*In vivo* (SD rats)	1 g/mL	Oral gavage paired with biodegradable chitin conduit bridging a 2mm sciatic nerve defect	Promoted peripheral nerve regeneration in rats with SNI (increased MNCV, fiber and axon diameter, g-ratio)	N/A	[85]
	Polysaccharides	*In vivo* (SD rats)	0.25 g/mL	Oral gavage	Promoted peripheral nerve regeneration in rats with sciatic nerve defect (increased SFI, TFI, PFI values, MNCV, and number of regenerated myelinated nerve fibers)	N/A	[86]
*Rhodiola rosea* L.	Salidroside	*In vivo* (SD rats)	5, 10 mg/kg	Intraperitoneal injection	Promoted peripheral nerve regeneration in rats with SNI (increased number and diameter of myelinated axons, number of motoneurons, SFI, amplitude of CMAP, MNCV)	N/A	[87]
*Scutellaria baicalensis* Georgi	Baicalin	*In vitro* (RSC96 SCs)	5, 10, 20 µM (dose-dependent manner)	Incubation	Promoted proliferation of RSC96 SCs	Modulation of neurotrophic factors (GDNF, BDNF, CNTF) and S100β	[88]
*Trigonella foenum-graecum* (fenugreek)	IND01 (Fenugreek seed extract)	*In vivo* (Wistar rats)	50, 100, 200 mg/kg	Oral administration	Promoted peripheral nerve regeneration in rats with: (1) partial sciatic nerve ligation (ameliorated thermal hyperalgesia, improved motor function test scores) (2) SNI (ameliorated thermal hyperalgesia, improved motor function test scores, increased MNCV)	N/A	[89]
*Tripterygium wilfordii* Hook. F.	Triptolide	*In vivo* (SD rats)	100 µg/kg	Intraperitoneal injection	Promoted peripheral nerve regeneration in rats with SNI (increased number of motoneurons, number of myelinated axons, diameter of nerve fibers, SFI, CMAP amplitude, MNCV, muscle fiber area)	Reduction of TNF-α, IL-β, and IL-6 expression	[90]
MUSHROOM							
*Amanita muscaria*	Muscimol	*In vivo* (SD rats)	400 μg/mL	Applied directly to the right L5 DRG	Promoted peripheral nerve regeneration in rats with SNI (prevented the development of thermal and mechanical hypersensitivity and mechanical allodynia, improved basal membrane integrity, and increased nerve fibers)	Normalization of PMP22 protein expression level by GABAergic modulation in the ipsilateral DRG	[91]
*Hericium erinaceus*	Aqueous extract	*In vivo* (SD rats)	10 mL/kg	Oral administration	Promoted peripheral nerve regeneration in rats following peroneal nerve crush	Activation of signaling pathways (Akt, MAPK, c-Jun, c-Fos) and protein synthesis	[92]
	Polysaccharide	*In vivo* (SD rats)	30 mg/mL/kg	Oral administration	Promoted sensory functional recovery following peroneal nerve crush in rats (reduced withdrawal reflex latency)	Activation of Akt and p38 MAPK signaling and increased expression of RECA-1	[93]
	Aqueous extract	*In vivo* (SD rats)	10, 20 mL/kg	Oral administration	Promoted peripheral nerve regeneration in rats following peroneal nerve crush (increased PFI, improved axon morphology, and development of neuromuscular junction)	N/A	[94]
*Lignosus rhinocerotis*	Aqueous extract	*In vivo* (SD rats)	500, 1000 mg/kg	Oral administration	Promoted motor and sensory functional recovery in rats with SNI (improved WRL and toe-spreading reflex)	N/A	[95]
DECOCTION							
Bogijetong	(1) Bogijietong decoction (18 ingredients) (2) A reconstituted formulation of BGJTD (BeD) with 4 ingredients (3) *Angelica gigas* (an ingredient in BeD)	*In vitro* (Primary neurons isolated from DRG at lumbar levels 4 and 5 in adult rats)	400 mg/kg	Incubation	Promoted neurite outgrowth of DRG neurons	(1) BGJTD and BeD: Downregulation of TNF-α and p38, upregulation of p-ERK1/2; (2) *Angelica gigas*: Regulation of ERK1/2 activity and TNF-α production	[96]
		*In vivo* (SD rats and BALB/c mice)	400 mg/kg	Oral administration	Reduced latency time in rats		
Buyang Huanwu	Buyang Huanwu decoction (16 ingredients: Modified formulation)	*In vivo* (SD rats)	1800 mg/kg	Oral administration paired with silicone rubber tube bridging a 10mm sciatic nerve defect	Promoted peripheral nerve regeneration in rats with sciatic nerve defect (increased nerve formation, myelinated axons, and endoneurial area)	N/A	[97]
Jiaweibugan	Jiaweibugan decoction (9 ingredients)	*In vivo* (Wistar rats)	28.6 g/kg	Intragastric administration	Protective effect on peripheral nerve injury by playing an anti-oxidative role in a diabetic rat model (increased MNCV and serum levels of glutathione, decreased serum levels of MDA)	Downregulation of NF-kB p65 and p38 MAPK mRNA expression	[98]
Qian-Zheng-San	Qian-Zheng-San (3 ingredients: *Typhonii rhizoma*, *Bombyx batryticatus*, *Scorpio*)	*In vivo* (SD rats)	1.75 g/mL	Oral gavage	Promoted peripheral nerve regeneration in rats with sciatic nerve defect (Increased SFI, MNCV, muscle wet weight, number of regenerated axons, axon diameter, nerve fiber diameter, myelin thickness, number of motor neurons in the lumbar spinal cord anterior horn)	N/A	[99]

Akt—protein kinase B; Bad—Bcl-2 associated agonist of cell death; Bax—Bcl-2-associated X protein; Bcl-2—B-cell lymphoma 2; BDNF—brain-derived neurotrophic factor; BGJTD—Bogijetong decoction; Cdc2—cell division cycle protein 2 homolog; CGRP—calcitonin gene-related peptide; CMAP—compound muscle action potential; CNTF—ciliary neurotrophic factor; CSA—cross-sectional area; DRG—dorsal root ganglion; DUOX2—dual oxidase 2; ERK—extracellular signal-regulated kinase; FGF—fibroblast growth factor; GABA—γ-aminobutyric acid; GAP-43—growth associated protein 43; GDNF—glial cell-derived neurotrophic factor; ICR mice—Institute of Cancer Research mice; IFN—interferon; IGF-1—insulin-like growth factor 1; IGF-IR—insulin-like growth factor 1 receptor; IL—interleukin; JNK—c-Jun N-terminal kinase; MAPK—mitogen-activated protein kinase; MBP—myelin basic protein; MDA—malondialdehyde; MMP—matrix metallopeptidase; MNCV—motor nerve conduction velocity; NF-κB—nuclear factor kappa B; NGF—nerve growth factor; NOX4—nicotinamide adenine dinucleotide phosphate oxidase 4; PAI-1—plasminogen activator inhibitor-1; PBS—phosphate buffered saline; PC12—pheochromocytoma cells; PCNA—proliferating cell nuclear antigen; PDGF—platelet-derived growth factor; PFI—peroneal nerve function index; PI3K—phosphoinositide 3-kinase; PMP22—peripheral myelin protein 22; Prom1—prominin 1; RECA-1—mouse monoclonal endothelial cell antibody; ROS—reactive oxygen species; RSC96 SC—RSC96 Schwann cell; SCs—Schwann cells; SD rats—Sprague-Dawley rats; SFI—sciatic function index; SNI—sciatic nerve injury; Sox18—sex-determining region Y-box transcription factor 18; TGF-β—transforming growth factor beta; TNF-α—tumor necrosis factor alpha; tPA—tissue plasminogen activator; Trk—tropomyosin receptor kinase; TRPC1—classical transient receptor potential 1; TSI—toe spread index; uPA—urokinase plasminogen activator; Vegf—vascular endothelial growth factor; WRL—withdrawal reflex latency.

### 4.4. Experimental Strategies and Neuroprotective Effects of Complementary and Alternative Medicines (CAMs) against Peripheral Nerve Injury

#### 4.4.1. CAMs with Neuroregenerative Potential

Due to the limitations of conventional therapies for PNIs, much attention has been dedicated to finding alternative approaches in treating PNIs. To date, studies have explored the potential of 20 species of plants, three species of mushrooms, and four types of decoctions in promoting peripheral nerve regeneration (Table 1). Notably, the neuroregenerative potential of *Achyranthes bidentata* [44,45,46,47,48,49], *Astragalus membranaceus* [52,53,54,55], *Curcuma longa* [60,61,62,63,64,65], *Panax ginseng* [73,74,75], and *Hericium erinaceus* [92,93,94] have been most studied. A total of 18 natural products have been identified across the studies, and their chemical structures are shown in Table 2. Among those, ursolic acid, syringic acid, and quercetin are the NPs that can be found across a variety of plant species [78,79,81,101,102,103]. Decoctions are usually made according to traditional formulae. However, among the decoctions discussed in this study, the Bogijetong decoction is a relatively modern formulation that was specifically developed to treat neuropathic pain [96].

#### 4.4.2. *In Vitro* Studies on Neuroregenerative Potential of CAMs

**Figure 4:**
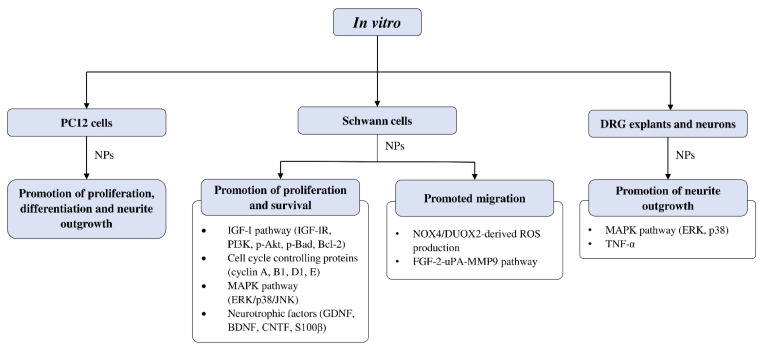
Overview of *in vitro* studies that demonstrated the effects of natural products relating to peripheral nerve regeneration across different cell types with associated mechanisms. Akt—protein kinase B; Bad—Bcl-2 associated agonist of cell death; Bcl-2—B-cell lymphoma 2; BDNF—brain-derived neurotrophic factor; CNTF—ciliary neurotrophic factor; DRG—dorsal root ganglion; DUOX2—dual oxidase 2; ERK—extracellular signal-regulated kinase; FGF—fibroblast growth factor; GDNF—glial cell-derived neurotrophic factor; IGF-I—insulin-like growth factor 1; IGF-IR—insulin-like growth factor 1 receptor; JNK—c-Jun N-terminal kinase; MAPK—mitogen-activated protein kinase; MMP9—matrix metallopeptidase 9; NOX4—nicotinamide adenine dinucleotide phosphate (NADPH) oxidase 4; NPs—natural products; PC12—pheochromocytoma cells; PI3K—phosphoinositide 3-kinase; ROS—reactive oxygen species; S100β—S100 calcium-binding protein β; TNF-α—tumor necrosis factor-α; uPA—urokinase plasminogen activator.

Figure 4 summarizes the *in vitro* studies on neuroregenerative properties of complementary and alternative medicines. Most of the studies were in Schwann cells, with a few using DRG explants, neurons, and PC12 cells (rat pheochromocytoma). Some CAMs were reported to induce proliferation, differentiation, and neurite outgrowth in PC12 cells. Similarly, neurite outgrowth was also promoted in DRG neurons through modulation of the extracellular signal-regulated kinase (ERK), p38, and tumor necrosis factor-α (TNF-α). Polypeptides isolated from *Achyranthes bidentata* have demonstrated the ability to promote neurite outgrowth in DRG neurons through the activation of ERK1/2 [45,46]. These findings resemble an earlier study that also reported neurite growth in DRG neurons induced by CD95 through ERK activation [104]. The Bogijetong decoction and its reconstituted formulation BeD elicited similar neuroprotective effects through downregulation of p38 and TNF-α [96] It was previously shown that TNF-α could inhibit neurite outgrowth in cultured DRG neurons [105,106], whereas the application of a TNF-α antagonist supported axonal regeneration following nerve injury [107].

##### Effects of CAMs on Schwann Cell Activity *In Vitro*

The studies examining the effects of complementary and alternative medicines and their related natural products on Schwann cells are primarily focused on promoting their proliferation and survival. The molecular mechanisms that were investigated in these studies include signaling pathways such as IGF-I and MAPK, as well as cell cycle controlling proteins and various neurotrophic factors (Figure 4). Past studies have demonstrated that ERK is required for proper myelination of SCs during development [108,109], and ERK signaling was rapidly activated following nerve injury, contributing to SC differentiation [110]. Moreover, evidence suggests that nerve regeneration following injury is closely associated with ERK [111,112], and ERK inhibition leads to impaired regenerative capability [111,113]. On the other hand, inhibition of p38 MAPK prevented SC demyelination and dedifferentiation, indicating its role in promoting the breakdown of myelin following nerve injury [114]. It is not unexpected that cyclins are associated with SC proliferation, as these proteins control cell cycle progression through the interaction of cyclin-dependent kinases. For instance, cyclin D is associated with Cdk4 or Cdk6 in the G1 phase, cyclin A participates with Cdk1 or Cdk2 in the S phase, cyclin E is involved with Cdk2 in G1 and S phases, cyclin B and Cdk1 regulates M phase [115,116].

Protocatechuic acid isolated from *Alpinia oxyphylla* Miq [50] and the aqueous extract of *Codonopsis pilosula* [58] were found to promote SCs proliferation by further enhancing IGF-I (insulin-like growth factor 1) signaling. The IGF-I growth factor is known to play a crucial role in neuromuscular recovery following injury. It is reported to be involved in promoting G1/S cell cycle progression [117] and survival of SCs [118] *in vitro*, and to facilitate peripheral nerve regeneration *in vivo* [119,120,121,122]. One study reported baicalin, a flavonoid that possesses various neuroprotective effects [123], induced proliferation of SCs through the modulation of neurotrophic factors including glial cell-derived neurotrophic factor (GDNF), BDNF, and ciliary neurotrophic factor (CTNF) [88]. These neurotrophic factors are integral to many aspects of nerve regeneration, as evident in past studies that showed their roles in myelin formation [124,125] and axonal regeneration [126,127].

In addition to promoting the proliferation of SCs, some NPs may promote the migratory ability of SCs, which is essential for regeneration and remyelination following nerve injury. Aqueous extracts of *Alpinia oxyphylla* Miq [51], *Astragalus membranaceus* [55], *Citrus medica* var. *sarcodactylis* [57], *Codonopsis pilosula* [58], and ginsenoside Rg1 isolated from ginseng [73] enhanced SC migration through the activation of FGF-2 signaling. The role of FGF-2 in the repair and regeneration of tissues [128] and its involvement in cell migration [129,130] is widely documented. A recently published study reported that another subfamily member, FGF5, is also involved in regulating SC migration and adhesion [131]. Besides FGF-2 signaling, another study investigating polypeptides of *A. bidentata* revealed that the upregulation of NOX4/DUOX2-derived reactive oxygen species (ROS) production was responsible for promoting SC migration [44]. Excessive accumulation of ROS production has been linked to neurodegenerative disorders [132] and peripheral neuropathy [133], but moderate levels of ROS may prove beneficial by acting as signal messengers in regulating biological processes, including cell adhesion and migration [134,135]. Syringic acid was shown to promote the proliferation and migration of SCs *in vitro*. Although the expression of several microRNAs was affected by syringic acid, further analysis suggested that the plant polyphenol promoted SC proliferation and migration mainly by suppressing the microRNA miR-451-5p [78].

#### 4.4.3. *In Vivo* Studies on Neuroregenerative Potential of CAMs

**Figure 5:**
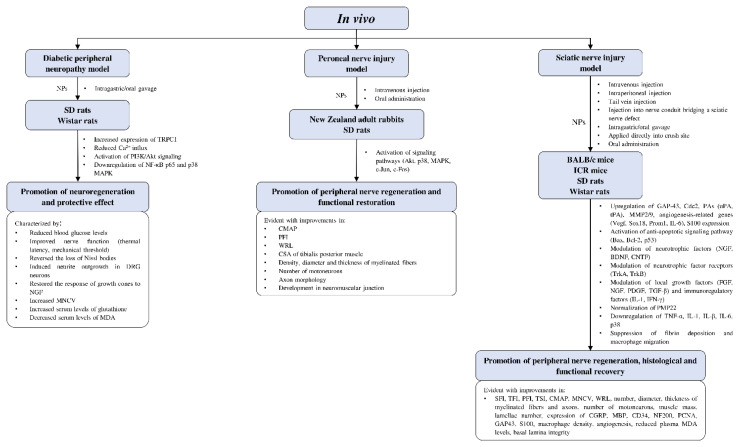
Overview of *in vivo* studies that demonstrated the effects of natural products relating to peripheral nerve regeneration across different experimental models with associated mechanisms. Akt—protein kinase B; Bax—Bcl-2-associated X protein; Bcl-2—B-cell lymphoma 2; BDNF—brain-derived neurotrophic factor; Cdc2—cell division control protein; CGRP—calcitonin gene-related peptide; CMAP—compound muscle action potential; CNTF—ciliary neurotrophic factor; CSA—cross-sectional area; DRG—dorsal root ganglion; FGF—fibroblast growth factor; GAP-43—growth associated protein 43; ICR—Institute of Cancer Research; IFN-γ—interferon-γ; IL—interleukin; MAPK—mitogen-activated protein kinase; MBP—myelin basic protein; MDA—malondialdehyde; MMP2/9—matrix-metalloproteinase-2/9; MNCV—motor nerve conduction velocity; NF-κB—nuclear factor kappa B; NGF—nerve growth factor; NPs—natural products; PAs—plasminogen activators; PCNA—proliferating cell nuclear antigen; PDGF—platelet-derived growth factor; PFI—peroneal function index; PI3K—phosphoinositide 3-kinase; PMP22—peripheral myelin protein 22; Prom1—prominin 1; SD—Sprague-Dawley; SFI—sciatic function index; Sox18—sex-determining region Y-box transcription factor 18; TFI—tibial function index; TGF-β—transforming growth factor-β; TNF-α—tumor necrosis factor-α; tPA—tissue plasminogen activator; Trk—tropomyosin receptor kinase; TRPC1—transient receptor potential cation channel subfamily C member 1; TSI—toe spread index; uPA—urokinase plasminogen activator; Vegf—vascular endothelial growth factor; WRL—withdrawal reflex latency.

Current *in vivo* studies on the potential of complementary and alternative medicines in peripheral nerve regeneration are limited to rodent models (Figure 5 and Table 1). Most of the studies involved different strains of rats and mice, with only two studies using rabbits as their animal models. Models of peripheral nerve injury used in the studies include diabetic peripheral neuropathy, peroneal nerve injury, and sciatic nerve injury. The effects of CAMs on peripheral nerve regeneration were evaluated by functional recovery indexes (e.g., PFI; sciatic function index, SFI; tibial function index, TFI; CMAP; MNCV; and WRL) and histological examinations (e.g., number, diameter, the thickness of myelinated fibers and regenerated axons; the number of motoneurons; and muscle mass).

##### Diabetic Peripheral Neuropathy Model

In the diabetic neuropathy (DPN) model, aqueous extract of Cortex Mori Radicis had anti-diabetic and neuroregenerative effects, as evidenced by reduced blood glucose levels, induced neurite outgrowth, restoration of the loss of Nissl bodies, and a response in the growth cones of DRG neurons [82]. The authors identified that the observed effects were mediated by the activation of the PI3K/Akt pathway and increased expression of TRPC1, which in turn reduced Ca2+ influx. The PI3K/Akt pathway is a crucial intracellular signaling pathway that governs cell proliferation, survival, and metabolism [136], its protective role against DPN has been previously hinted at [137,138]. The transient receptor potential (TRPC) is a family of Ca2+-permeable channels that participates in axonal regeneration [139]. In particular, TRPC1 and TRPC4 were shown to induce neurite outgrowth in PC12 cells and DRG neurons [140,141]. In another study, administration of Jiaweibugan decoction in a DPN model ameliorated changes in motor nerve conduction velocity (MNCV), and malondialdehyde (MDA), and glutathione levels through an anti-oxidative pathway via downregulating NF-κB p65 and p38 MAPK [98]. The activation of p38 MAPK, which belongs to a family of kinases that are responsive to stress stimuli, further activates NF-κB leading to inflammation, a driving factor of DPN [142,143].

##### Peroneal Nerve Injury Model

In the peroneal nerve injury model, aqueous extract and polypeptides of *A. bidentata* were shown to enhance nerve regeneration [45,49], as indicated by increased density and diameter of myelinated fibers, and numbers of motor neurons. Although behavioral analyses were not performed in the studies, improvements in compound muscle action potential (CAMP) demonstrated the ability of *A. bidentata* aqueous extract and polypeptides to promote functional recovery. Aqueous extract and polysaccharides from *Hericium erinaceus* also exhibited nerve regeneration and functional recovery following peroneal nerve crush [92,93,94], as evidenced by the improvements in the peroneal function index (PFI), withdrawal reflex latency (WRL) and axon morphology, and the development of neuromuscular junction. These findings were supported by the activation of Akt, p38, c-Jun, and c-Fos, which is in line with other studies that showed the importance of these signaling events for axonal regeneration [144,145,146].

##### Sciatic Nerve Injury Model

The sciatic nerve injury (SNI) model is the most commonly used model in the study of the effects of complementary and alternative medicines on peripheral nerve regeneration, and many studies have investigated the underlying mechanisms or molecular pathways through which CAMs elicit their neuroregenerative properties. For instance, polypeptides of *A. bidentata* [47], astragaloside IV isolated from *A. membranaceus* [52], and aqueous extract of *Dioscoreae rhizoma* [66] promoted nerve regeneration via upregulation of GAP-43 expression. The GAP-43 protein is highly associated with the development and plasticity of the nervous system [147]. Its expression is known to be elevated following nerve injury [148] and is involved in the neurite outgrowth of hippocampal neurons [149]. Similarly, modulation of other neurotrophic factors such as NGF, BDNF, CNTF [47,54], and pro-inflammatory cytokines including IL-1, IL-6, IL-β, and TNF-α [54,90] were also involved in promoting nerve regeneration as well. Although an inflammatory response following injury is necessary for the regenerative process [150], prolonged inflammation can impede recovery and may even lead to the development of neuropathic pain [151], which reflects the double-edged property of inflammation and the importance of proper regulation. Additionally, a study on *Ginkgo biloba* extract showed that it promoted axonal angiogenesis through the modulation of related genes, including Vegf, Sox18, Prom1, and IL-6 [71]. Studies have also demonstrated the participation of Vegf [152,153], Prom1 [154], and another subfamily gene, Sox11 in sciatic nerve regeneration [155], and the restorative role of IL-6 has also been implied in DPN and central nervous system models [156,157]. Muscimol prevented hyperalgesia through the modulation of PMP22 [91], a key component of the basal lamina. The expression of PMP22 is correlated with myelin formation and nerve regeneration [158,159]. Studies investigating EGCG in an SNI model showed that it had neuroprotective and regenerative effects, partly due to the modulation of the apoptotic machinery, including Bax, Bcl-2, p53, and survivin [76,77]. The subsequent loss of neurons after PNI is known to be closely related to apoptosis [160] which is partly influenced by p53 and Bax [161], while the association of survivin in nerve injury has also been documented [162].

#### 4.4.4. Involvement of CAMs in Combinatorial Approaches for the Treatment of PNI

There is increasing evidence that the successful repair and regeneration of nerves will require not just a single treatment strategy, but a multifaceted strategy involving different disciplines. Studies adopting combinatorial approaches have yielded interesting findings. For example, *Lycium barbarum* polysaccharide incorporated into core-shell structured nanofibrous scaffolds by coaxial electrospinning showed proliferative effects in PC12, SCs, and DRG neurons [80]. In two separate studies, puerarin, the active component extracted from *Pueraria lobata* roots, as well as rat serum metabolites of *P. lobata* enhanced the neuroregenerative effects of silicone rubber nerve chambers. Increases in myelinated axons and structurally mature regenerated axons were observed, while muscle reinnervation led to functional recovery, as indicated by an increase in action potential and nerve conduction [83,84]. Similar results were obtained with Buyang Huanwu decoction being administered as a co-treatment alongside silicone rubber nerve chambers, which led to more prominent axonal regeneration [97]. In an SNI model, a magnetic nanocomposite scaffold produced from using magnetic nanoparticles and biodegradable chitosan-glycerophosphate polymer enhanced SC viability, nerve regeneration, and functional recovery when paired with an applied magnetic field [163]. The use of nerve guiding conduits gained popularity over the years. They have been used to isolate regenerating axons from fibrotic tissues, to protect them from mechanical forces, and to guide new-forming tissue as well as condensing growth factors secreted by SCs [164]. The concept was initiated with a simple hollow design but has since advanced to innovative ways of redesigning nerve conduits to further extend their original capabilities 11. The attractive characteristics of modern nerve conduits offer tremendous potentials. These nerve conduits are occasionally paired with other strategies for improving nerve outcomes. For instance, Chang et al. [165] developed a natural biodegradable multi-channeled scaffold with aligned electrospun nanofibers and a neurotrophic gradient, which resulted in superior nerve recovery and less muscle atrophy compared with nerve autografts. Hussin et al. [56] used *Centella asiatica* (L.) to neurodifferentiate mesenchymal stem cells. This was subsequently developed with decellularized artery as a nerve conduit, which demonstrated functional restoration in an SNI model similar to that of reversed autograft.

### 4.5. Limitations and Future Prospects

**Figure 6:**
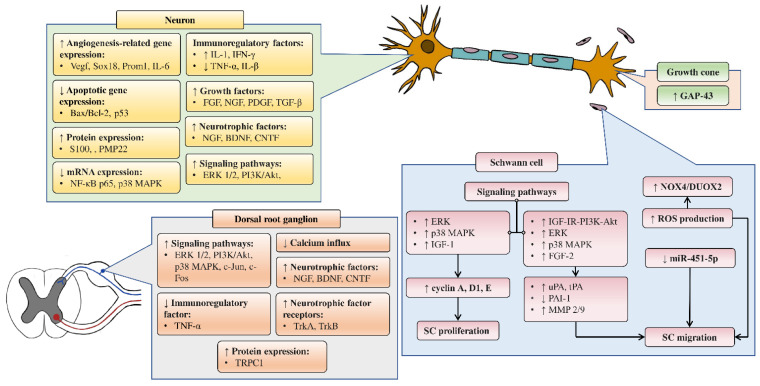
Summary of the molecular mechanisms associated with the neuroregenerative effects of CAMs. Vegf—vascular endothelial growth factor; Sox18—sex-determining region Y-box transcription factor 18; Prom1—prominin 1; IL—interleukin; IFN-γ—interferon-γ; Bax—Bcl-2-associated X protein; Bcl-2—B-cell lymphoma 2; Trk—tropomyosin receptor kinase; PMP22—peripheral myelin protein 22; FGF—fibroblast growth factor; NGF—nerve growth factor; PDGF—platelet-derived growth factor; TGF-β—transforming growth factor-β; NF-κB—nuclear factor kappa B; MAPK—mitogen-activated protein kinase; PI3K—phosphoinositide 3-kinase; Akt—protein kinase B; BDNF—brain-derived neurotrophic factor; CNTF—ciliary neurotrophic factor; TNF-α—tumor necrosis factor-α; TRPC1—transient receptor potential cation channel subfamily C member 1; GAP-43—growth-associated protein 43; PAI-1—plasminogen activator inhibitor-1; MMP2/9—matrix-metalloproteinase-2/9; tPA—tissue plasminogen activator; uPA—urokinase plasminogen activator.

As mentioned earlier, PNI represents a significant health issue while the effectiveness of current treatment approaches is highly subjective. Hence, substantial effort is required to discover and establish proper methods for the management of PNI. Present studies have shown promising findings in utilizing various applications including nerve conduits [166], stem cell therapy [167], phytochemicals [22], and electrical stimulation [168] for treating PNI, and their potential may subsequently be improved when paired together. Evidence from *in vitro* and *in vivo* studies have delineated the neuroregenerative properties of various CAMs, and the underlying mechanisms have been investigated (as summarized in Figure 6), although they still remain incompletely understood and require further elucidation. Subsequently, pre-clinical and clinical studies on existing potential candidates and approaches should be supported to drive the development of future therapeutics.

Existing studies on the effect of complementary medicines in treating PNI are preliminary findings with limited information (Table 1). The majority of studies investigated crude extracts or specific fractions of extracts, with only 24 out of the 56 studies managing to identify the exact NPs responsible for the observed effects. Additionally, 25 studies did not report the underlying mechanisms for the resultant effects of NPs, especially at the *in vivo* stage. This situation highlights the need for greater efforts among the scientific community to fully investigate the purported effects of NPs. Another issue is the route and method of administration *in vivo*. It is known that oral administration is generally economical and relatively safe, but the resultant efficacy may not be reliable due to uncontrollable animal habits and behaviors [169]. In contrast, gavage or injection routes typically require some form of restraint, which may result in animal stress that may influence study outcomes. The administration routine also varied across studies, with the treatments lasting from a few days to months. Moreover, treatment frequency also influences experimental outcomes. Although it is difficult to standardize animal handling procedures, these factors should be taken into account with carefully designed studies.

In this review, the majority of studies on NPs as a treatment for PNI were based on plants and herbs, with a few studies on mushrooms such as *Amanita muscaria*, *Hericium erinaceus*, and *Lignosus rhinocerotis*, as well as some decoctions. This is unsurprising, considering that phytochemicals are highly studied for drug development, which should shed more light on this area of research [170,171,172]. However, the use of NPs for peripheral nerve repair and regeneration is still largely overlooked and could be an untapped potential source for promising drug candidates. For instance, a previous study demonstrated that various mushrooms including *Agaricus blazei* Murrill, *Antrodia cinnamomea*, *Ganoderma lucidum*, and *Hirsutella sinensis* could activate intracellular signaling kinases ERK, JNK, and p38, which are associated with peripheral nerve regeneration [173]. Another study showed that *G. lucidum*, *Ganoderma neo-japonicum*, and *Grifola frondosa* promoted neuritogenesis via the involvement of the MAPK signaling pathway [174]. Aside from exploring untapped sources of NPs, future research may also simultaneously examine the efficiency of CAMs or NPs with known neuroregenerative properties to compare their ability to promote regeneration of peripheral nerves.

The use of algae in peripheral nerve regeneration merits attention. Algae are well-known for their diverse applications in food nutrition [175], biofuels [176], cosmetics [177], and pharmaceuticals [178,179]. Recent studies have also demonstrated that algae could have potential in the treatment of neurological disorders, including Parkinson’s and Alzheimer’s disease [180,181]. However, the potential uses of algae in peripheral nerve regeneration have yet to be explored, despite evidence showing the ability of macroalgae to promote neurite outgrowth in hippocampal neurons [182,183,184]. More recently, a study showed that *Gracilaria manilaensis* induced the proliferation of neurite-bearing cells in the rat pheochromocytoma cell line, which is believed to mimic the neuroactivity of NGF [185]. Thus, investigation on the nerve regenerative potential of other NPs holds much promise.

## 5. Conclusions

Peripheral nerve injury remains a challenge, while future prospects are leaning towards multi-combinatorial approaches. Natural products are highly appreciated for their therapeutic value, and there is a growing body of evidence in their potential for peripheral nerve regeneration. The present findings showed that various NPs promote the proliferation and migration of SCs, most commonly through the activation of MAPK and FGF-2 signaling pathways, respectively. Promotion of peripheral nerve regeneration was also observed in rodent models, partly through the modulation of neurotrophic factors, pro-inflammatory cytokines, and anti-apoptotic signaling. Hence, NPs could play key roles in nerve repair and regeneration in the near future, especially when paired with other innovative approaches such as modern nerve conduits.

## Figures and Tables

**Table 2:** Chemical structures of natural products and their respective sources.

Sources	Natural Product	Chemical Structure
Alpinate Oxyphyllae Fructus (*Alpinia oxyphylla* Miq)	Protocatechuic acid	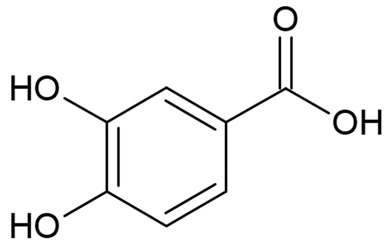
*Astragalus membranaceus*	Astragaloside IV	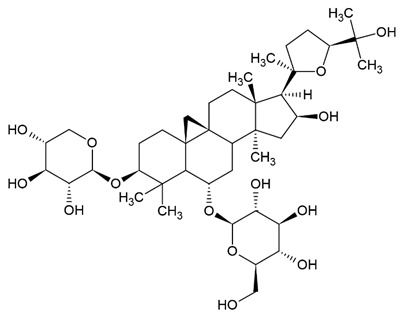
*Crocus sativus*	Crocin	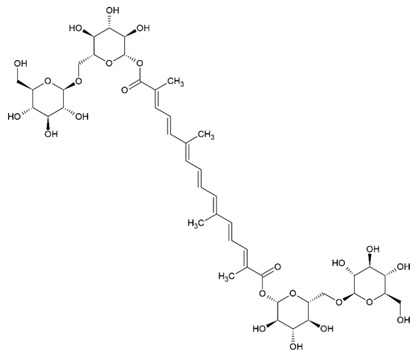
*Curcuma longa*	Curcumin	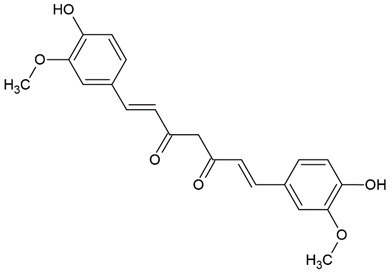
*Epimedium*	Icariin	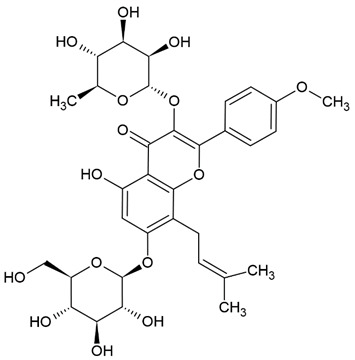
*Gardenia jasminoides* Ellis	Genipin	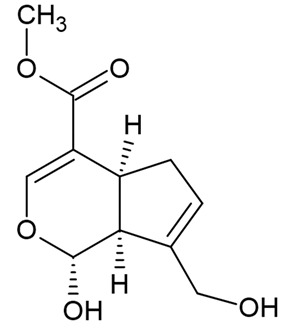
*Gastrodia elata* Blume	Gastrodin	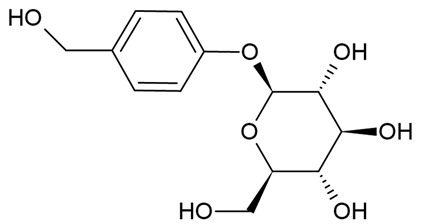
Ginseng	Ginsenoside Rg1	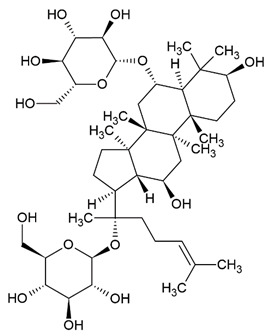
	Ginsenoside Re	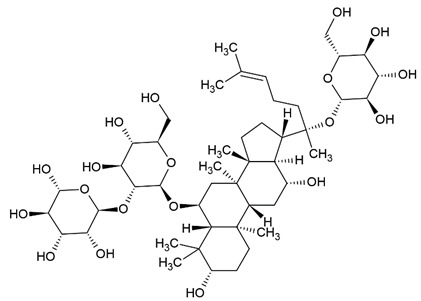
Green tea	(-)-Epigallocatechin-3-gallate (EGCG)	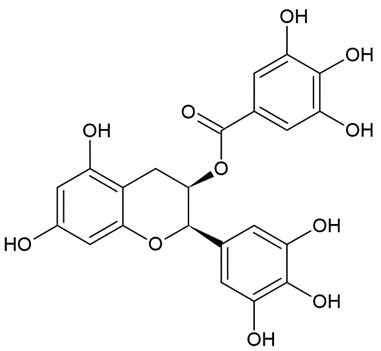
Isolated from a variety of plants (e.g., grapes, *Vitis vinifera*; olive, *Olea europaea*; radish, *Raphanus sativus*; pumpkin, *Cucurbita pepo* [101])	Syringic acid	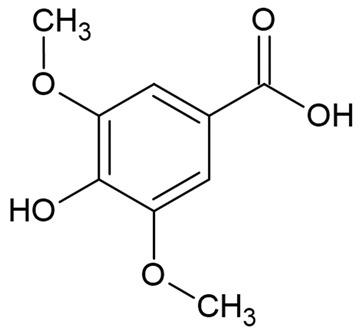
Isolated from a variety of plants (e.g., apple, *Malus domestica*; cranberry, *Vaccinium oxycoccus*; peppermint, *Mentha piperita*; and thyme, *Thymus vulgaris* [102])	Ursolic acid	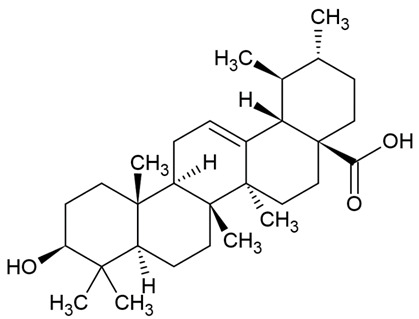
Isolated from a variety of plants (e.g., apple, *Malus domestica*; caper, *Capparis spinosa*; onion, *Allium cepa*; tomato, *Solanum lycopersicum*; and grapes, *Vitis vinifera* [103])	Quercetin	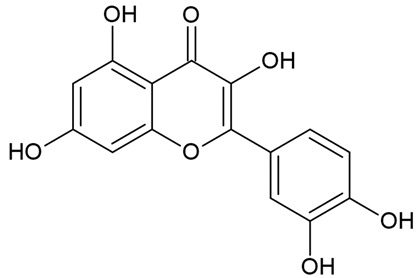
Pueraria lobata	Puerarin	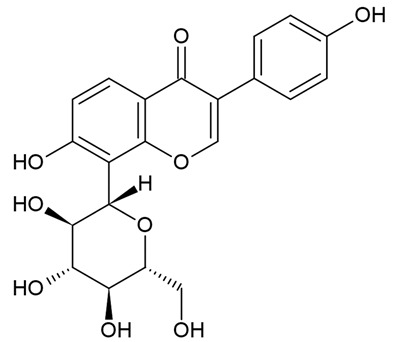
*Rhodiola rosea* L.	Salidroside	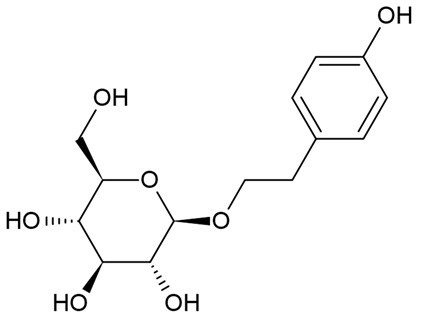
*Scutellaria baicalensis* Georgi	Baicalin	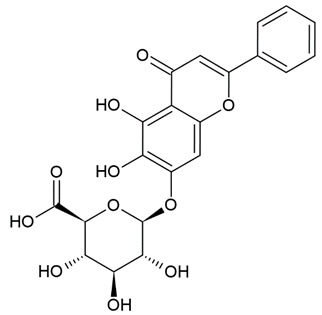
*Tripterygium wilfordii* Hook.F.	Triptolide	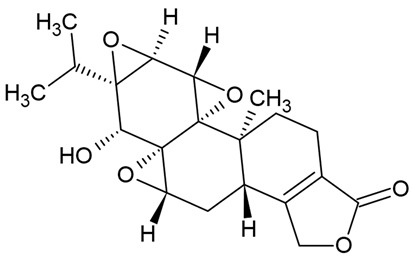
*Amanita muscaria*	Muscimol